# Neuronal Protection by Ha-RAS-GTPase Signaling through Selective Downregulation of Plasmalemmal Voltage-Dependent Anion Channel-1

**DOI:** 10.3390/ijms25053030

**Published:** 2024-03-06

**Authors:** Sebastian Neumann, Konstantin Kuteykin-Teplyakov, Rolf Heumann

**Affiliations:** Department of Biochemistry II—Molecular Neurobiochemistry, Faculty of Chemistry and Biochemistry, Ruhr-Universität Bochum, 44801 Bochum, Germany; konstantin.neurocreate@gmail.com

**Keywords:** RAS-GTPase, neuroprotection, signal transduction, transgenic mouse, VDAC-1

## Abstract

The small GTPase RAS acts as a plasma membrane-anchored intracellular neurotrophin counteracting neuronal degeneration in the brain, but the underlying molecular mechanisms are largely unknown. In transgenic mice expressing constitutively activated V12-Ha-RAS selectively in neurons, proteome analysis uncovered a 70% decrease in voltage-dependent anion channel-1 (VDAC-1) in the cortex and hippocampus. We observed a corresponding reduction in the levels of mRNA splicing variant coding for plasma membrane-targeted VDAC-1 (pl-VDAC-1) while mRNA levels for mitochondrial membrane VDAC-1 (mt-VDAC-1) remained constant. In primary cortical neurons derived from V12-Ha-RAS animals, a decrease in pl-VDAC-1 mRNA levels was observed, accompanied by a concomitant reduction in the ferricyanide reductase activity associated with VDAC-1 protein. Application of MEK inhibitor U0126 to transgenic cortical neurons reconstituted pl-VDAC-1 mRNA to reach wild-type levels. Excitotoxic glutamate-induced cell death was strongly attenuated in transgenic V12-Ha-RAS overexpressing cortical cultures. Consistently, a neuroprotective effect could also be achieved in wild-type cortical cultures by the extracellular application of channel-blocking antibody targeting the N-terminus of VDAC-1. These results may encourage novel therapeutic approaches toward blocking pl-VDAC-1 by monoclonal antibody targeting for complementary treatments in transplantation and neurodegenerative disease.

## 1. Introduction

Neurotrophins consist of a family of extracellular proteins promoting the differentiation, growth, survival, and connectivity of brain neurons during development and in the adult stage [1]. Neurotrophins and their peptide mimetics are expected to counteract the devastating effects of neurodegenerative disorders of the central nervous system (CNS), involving those of Alzheimer’s disease (AD) or Parkinson’s disease (PD) [2,3], but the development of clinical treatments was not successful in previous attempts [4]. Thus, new therapeutic approaches may evolve from a detailed knowledge of the intracellular signal transduction pathways activated by neurotrophins. 

In neurons, Ha-RAS proto-oncogene activity was found to be a switch for typical neurotrophic signaling effects such as the regulation of neurite growth and promotion of neuronal survival [5]. The evolutionary conserved intracellular signal protein, Rat sarcoma (RAS), belongs to the family of small monomeric guanosine triphosphatases (GTPases), which are anchored to the cytoplasmic face of the plasma membrane via the palmitoylation and farnesylation of cysteines at its C-terminal end. Typically, RAS becomes activated via receptor tyrosine kinases and the SHC-GRB2-SOS complex. Thereby, RAS functions as a molecular switch cycling between inactive guanosine diphosphate (GDP)-bound and signaling-active guanosine triphosphate (GTP)-bound conformation. The relative levels of GTP-bound RAS are enhanced (upregulated) by the guanine–nucleotide exchange factors (GEF) and decreased (downregulated) by GTPase-activating proteins (GAP) [6,7,8]. The RAS family comprises four highly conserved isoforms such as Harvey–RAS (Ha-RAS), Neuroblastoma–RAS (N-RAS), and Kirsten–RAS (K-RAS), which is alternatively spliced into K-RAS 4A and K-RAS 4B [6]. RAS activates several downstream signal transduction pathways, such as the RAF/MEK/ERK cascade, PI3-kinase (PI3K), TIAM1/RAC, RAL-GEF/RAL, and more [7]. 

To investigate the role of lifelong persistent RAS upregulation in a neuronal context, Heumann et al. established the transgenic mouse model (synRas) in which the rat synapsin I promoter restricts the expression of constitutively activated human Ha-RAS oncogene (V12-Ha-RAS) to postmitotic neurons without the generation of any tumor. In these mice, the endogenous pan (Ha-, N-, and K-) RAS activity continues to be regulated by normal physiological stimuli during development and in adult animals [9]. V12-Ha-RAS protein is constitutively activated in neurons, as the bound GTP is hydrolyzed at a 5-fold reduced rate [10,11], resulting in a relative enhancement of the downstream endogenous mitogen-activated protein kinase (MAPK) activity [9,12]. 

One outstanding feature of synRas mice brain is enhanced neuronal protection after an injury as (i) motor neurons were protected from facial nerve lesions, (ii) neurotoxin 1-methyl-4-phenyl-1,2,3,6-tetrahydropyridin (MPTP)-induced degeneration was attenuated in dopaminergic neurons [9], (iii) differentiated cultures of synRas-derived neurospheres of the ventral mesencephalon revealed protection from the neurotoxin 6-hydroxydopamine (6-OHDA)-induced death [13], and (iv) hyperoxia-induced brain neurons were protected [14], which caused even secondary protection in the surrounding myelinating oligodendrocytes [15]. 

In order to gain further mechanistic insight, we thought to obtain candidate proteins involved in V12-Ha-RAS-mediated neuronal protection. Thus, we applied an unbiased proteome approach and checked the relevance of the data obtained toward the phenotype of synRas animals. We then decided to select voltage-dependent anion channel-1 (VDAC-1) for a detailed investigation, which was previously shown to be a pro-apoptotic membrane protein [16]. 

The three isoforms VDAC-1, VDAC-2, and VDAC-3 comprise the VDAC superfamily, differing in their tissue distribution and expression levels [17]. The most abundant isoform is VDAC-1, which is typically found in the outer mitochondrial membrane (mt-VDAC-1). Here, VDAC-1 enables the exchange of ATP/ADP, ions, and metabolites between the mitochondria and the cytosol [18]. In 2008, the structures of human and murine VDAC-1 were solved, revealing a β-barrel structure of 19 anti-parallel β-sheets and an N-terminal α-helix [19,20,21]. VDAC functions as a monomeric channel; however, there have been reports of VDAC oligomers, which are involved in mitochondria-mediated apoptosis [22]. 

Besides the mitochondria, the VDAC protein can be found in other subcellular locations, such as in the plasma membrane (pl-VDAC-1) [23,24,25], endoplasmic reticulum, and sarcoplasmic reticulum [26]. In mice, due to alternative splicing of the first exon of the VDAC-1 pre-mRNA, two different mRNA splice variants are generated, one coding for the mt-VDAC-1 and a second one for the pl-VDAC-1 [24,27]. A short hydrophobic signal peptide of 13 amino acids guides the pl-VDAC-1 protein through the secretory pathway to the plasma membrane. Before the pl-VDAC-1 becomes inserted into the plasma membrane, the signal peptide is cleaved off. Accordingly, there is no difference in the amino acid sequence between the mt-VDAC-1 and the pl-VDAC-1 [27]. Although the existence of pl-VDAC-1 protein is described for other species like humans or rats, alternative splicing to guide VDAC-1 to the plasma membrane has so far been exclusively described for mice [25]. The physiological role of pl-VDAC-1 is not completely understood yet; nevertheless, pl-VDAC-1 works as an NADH–ferricyanide reductase regulating normal redox homeostasis [28,29].

Interestingly, not only mt-VDAC-1 but also pl-VDAC-1 participates in apoptosis. Under apoptotic conditions, pl-VDAC-1 becomes activated, and the channel opens, whereas its blockage, e.g., due to the extra-cellular application of anti-VDAC-1 antibodies, prevents apoptosis [30,31,32]. In post mortem brains of Alzheimer’s disease patients, VDAC-1 is overexpressed [33], and therefore, prodromal Alzheimer’s disease might be recognized by VDAC-1 overexpression as a biomarker [34]. There is broad evidence showing the participation of VDAC-1 in Parkinson’s disease via its interaction with α-synuclein mediating its transport via the pore-regulating Ca^2+^-homeostasis [35], altogether providing evidence for the involvement of VDAC-1 in neurodegenerative diseases [34,36]. 

In this study, we explore Ha-RAS-mediated mechanism signaling for persistent neuroprotection in the degenerating adult brain by focusing on the role of pl-VDAC-1. 

## 2. Results 

### 2.1. Differential Brain Proteome Analysis between Wild-Type and synRas Mice

Although previous investigations have shown that the neuronal activation of Ha-RAS leads to neuroprotection [9,13,14,15,37,38], its molecular mechanism of signaling remains elusive. Here, we studied the proteome approach as follows: mice brains of wild-type (WT) and synRas animals were prepared from mitochondrial (M), synaptosomal (S), and cytosolic I fractions. Wild-type samples were labeled with the fluorophore Cyanine3 (Cy3) and the synRas-derived ones with Cyanine5 (Cy5). Samples of each fraction were pooled and separated by means of two-dimensional (2D) electrophoresis. The silver-stained 2D gels (Figure S1) show differentially regulated protein spots in each of the fractions. The 2D gels were scanned after exciting Cy3 as well as Cy5 (at 532 nm and 633 nm, respectively). The overlay image demonstrates differences in the protein expression and regulation between wild-type and synRas samples (Figure S2). In order to identify which of the proteins differ in their expression level, these spots (Figure S3) were cut out from the 2D gel and further characterized by MALDI-TOF analysis. 

The changes in the proteome can be categorized into upregulated (*n* = 9), downregulated proteins (*n* = 3), and modified proteins (*n* = 6) by comparing alterations between synRas-derived fractions to wild-type ones (Table 1). 

### 2.2. Transgenic Activation of RAS in Neurons Results in Downregulation of VDAC-1

In order to confirm the proteome results (Table 1), Western blot analysis was applied for selected proteins differing in their expression level between wild-type and synRas brain. The upregulation of Cytochrome C (mitochondrial fraction) (spot M247), ATP synthase β (spot M257), and Amphiphysin (spot S1), as found in proteomics (Table 1), could be confirmed by Western blot analysis (Table 1 and Table S1, Figure S4). As the proteome results demonstrated the appearance of ATP synthase α (spots M219, M220, M221; Table 1), the Western blot analysis displayed an upregulation for the ATP synthase α between wild type versus synRas (Table 1 and Table S1, Figure S4). Furthermore, the Western blot analysis confirmed the downregulation of VDAC-1 (spots M216, M217) and Stathmin (spot C120) (Table 1 and Table S1, Figure S4), as already revealed by proteomic analysis (Table 1). Synapsin I is not regulated in its native non-phosphorylated state, but using anti-phospho-4/5-synapsin I [G526] or anti-phospho-6-synapsin I [G555] antibodies, an upregulation was found in both cases for the synRas-derived samples (Table S1, Figure S4). Cytochrome C of the mitochondrial fraction showed a +1.23 ± 0.13 (*t*-test: *: *p* < 0.05) fold upregulation, while a −1.25 ± 0.25 (*t*-test: NS: not significant) fold downregulation of Cytochrome C of the cytosolic fraction was found by Western blot analysis (Table S1, Figure S4). The VDAC-1 was chosen for detailed investigation of these differentially regulated proteins.

### 2.3. Plasmalemmal-VDAC-1 but Not Mitochondrial-VDAC-1 mRNA Is Downregulated by the Transgenic Activation of Neuronal Ha-RAS 

**Figure 1:**
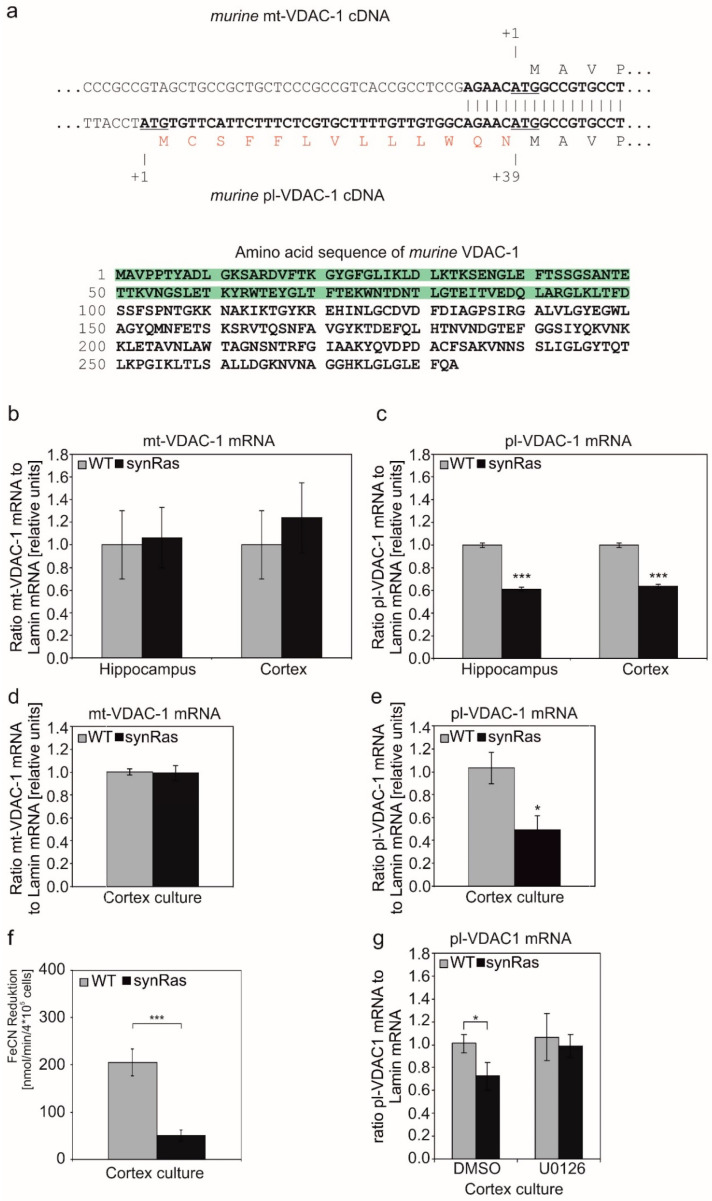
(**a**) The cDNA alignment (upper panel) shows the differences between mt-VDAC-1 and pl-VDAC-1 along with the endoplasmic reticulum (ER)-cleavable amino acid sequence in the one-letter code (red letters). The sequence was modified from [27]. The ATG start codon is underlined. In the lower panel, the full amino acid sequence of the murine VDAC-1 is shown (source: NCBI accession no. NM_011694). The first N-terminal 100 amino acids are highlighted in green as an antibody is directed against these amino acids. This antibody is used in experiments, which are described in 2.6 (see below). (**b**,**c**) Quantification of mt-VDAC-1 mRNA (**b**) and pl-VDAC-1 mRNA (**c**) in hippocampi and cortices and of adult WT and synRas mice. (**d**,**e**) Quantification of mt-VDAC-1 mRNA (**d**) and pl-VDAC-1 mRNA (**e**) in primary cortical cultures of wild-type and synRas mice. (**f**) NADH–ferricyanide reductase activity of wild-type and synRas primary cortical cultures. (**g**) The medium of wild-type and synRas-mice-derived cortical cultures were supplemented with 10 µM of MEK inhibitor U0126 to suppress the RAS-transgene enhanced MAPK pathway. After 3 days, the mRNA level of pl-VDAC-1 was quantified by RT-PCR. In all diagrams (**b**–**g**), bars show mean ± SEM derived from three (**b**–**f**) or four (**g**) independent experiments, in which each sample was measured as duplicate (*t*-test: *: *p* < 0.05, ***: *p* < 0.001).

As mentioned before, there are splicing variants in mice that code for mRNAs with or without a signal peptide (Figure 1a) [27]. Thus, we elucidated if there is a differential regulation of VDAC-1 splicing variants in synRas mice expressing constitutively activated V12-Ha-RAS in neurons. Splicing variant-specific primers were used for quantifying mt-VDAC-1 and pl-VDAC-1 mRNA levels by quantitative real-time PCR analysis in cortices and hippocampi of adult wild-type and synRas mice (Figure 1b,c). There was a selective and significant downregulation of the pl-VDAC-1 mRNA to 61 ± 2% or to 63 ± 2% found in hippocampi or cortices, respectively, in adult synRas mice while levels of mt-VDAC-1 mRNA remained constant. Consistently, in cortical primary cultures derived from synRas mice, there was a selective downregulation of pl-VDAC-1 mRNA to even 50 ± 11% in comparison to wild-type ones (Figure 1e), while the mt-VDAC-1 mRNA levels were unchanged (Figure 1d).

### 2.4. Transgenic Activation of Neuronal Ha-RAS in Cortical Neurons Resulted in Reduced pl-VDAC-1-Associated Ferricyanide Reductase Activity

Next, we asked if the downregulation of pl-VDAC-1 in synRas mice is reflected by a reduction in outer cell membrane-associated protein activity. Interestingly, pl-VDAC-1 was previously described to have an enzymatic activity as NADH–ferricyanide reductase for as yet unknown reasons [28,29]. As Akanda et al. reported how to measure the ferricyanide reductase cell surface activity in primary hippocampal neurons [31], we determined the NADH–ferricyanide reductase cell surface activity of wild-type and synRas-derived cortical cultures, accordingly. Reductase activity was reduced from 203 ± 28 nmol/min/4 × 10^5^ cells in wild type to 50 ± 12 nmol/min/4 × 10^5^ cells in synRas-derived cortical cultures (Figure 1f). 

### 2.5. Pharmacological Inhibition of MEK Prevents Downregulation of pl-VDAC-1 in Cortical Neuron Cultures

The obtained results led to the question of whether the observed downregulation of pl-VDAC-1 in synRas mice is mediated by the enhanced MAPK activity found in cortical neurons [9]. In order to address this question, we used the MEK inhibitor U0126 at a concentration of 10 µM, selectively targeting the proportion of MAPK activity that was over-activated by the RAS-transgene while the endogenous MAPK signaling was kept unchanged [40]. The mRNA was isolated from wild-type and synRas-derived cortical cultures that were treated with or without 10 µM U0126 for three days and subjected to quantitative real-time PCR analysis for analyzing the pl-VDAC-1 mRNA level.

A significant downregulation of pl-VDAC-1 mRNA was found for synRas cortical cultures (72.8 ± 12.2%) in untreated control cultures (Figure 1g), while in U0126-treated cultures, no differences in the pl-VDAC-1 mRNA levels were found (Figure 1g), indicating that the partial MAPK inhibition restored the pl-VDAC-1 mRNA level of synRas-derived cortical cells to the level of wild-type cortical cells. This shows that the RAS-transgene-mediated over-activated MAPK signaling in synRas mice changes the alternative splicing of the VDAC-1 gene.

### 2.6. Inhibition of pl-VDAC-1 by Blocking Antibody Mimics Protection from Glutamate-Induced Cell Death Found by Transgenic Activation of Ha-RAS

**Figure 2:**
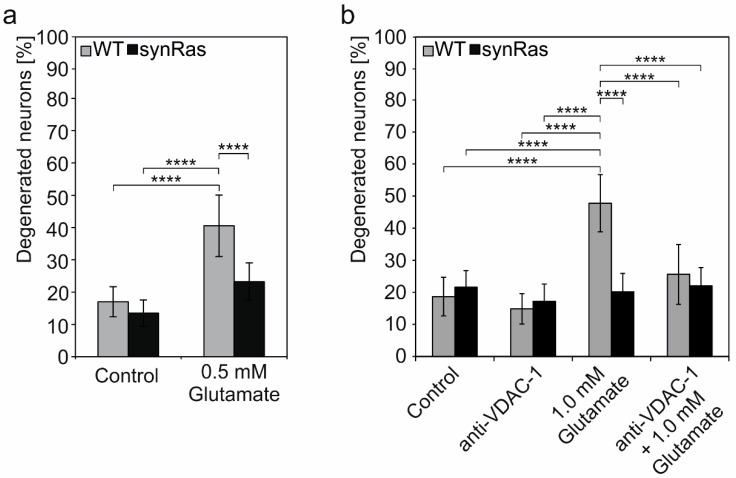
(**a**) Primary cortical cultures of wild-type and synRas mice were stimulated with 0.5 mM glutamate for 90 min. Neuronal survival was determined by trypan blue exclusion assay 24 h after excitotoxic stimulation. Bars represent the ratio of degenerated neurons to the total number of neurons as mean ± SEM out of at least four up to seven different randomly selected areas per condition from three independent experiments. In sum, 626 to 1050 neurons were analyzed (one-way ANOVA followed by Tukey post hoc test: ****: *p* < 0.0001). (**b**) Quantification of degenerated neurons in primary cortical of wild-type and synRas mice. Cells were pre-incubated without or with anti-VDAC-1 for one hour, followed by excitotoxic stimulation with 1 mM glutamate for 60 min. The number of degenerated neurons was quantified using the trypan blue exclusion assay after 24 h. Bars represent the ratio of degenerated neurons to the total number of neurons out of five different randomly selected areas per condition from four independent experiments. In total, 518 up to 822 neurons were analyzed per condition (one-way ANOVA followed by Tukey post hoc test: ****: *p* < 0.0001).

Next, synRas-derived primary cortical cultures were tested regarding their neuroprotective phenotype using excitotoxic glutamate-induced cell death models. Wild-type and synRas cultures were stimulated with 0.5 mM glutamate for 90 min, and the number of degenerated neurons was quantified by trypan blue exclusion assay 24 h after excitotoxic stimulation. Control cultures without glutamate stimulation showed a background of 13.4–17.0% degenerated neurons with no significant difference between wild-type and synRas neurons (Figure 2a). After excitotoxic stimulation with 0.5 mM glutamate, we observed 40.5 ± 9.5% degenerating wild-type neurons while only 23.3 ± 5.8% synRas-derived neurons showed degeneration resulting in a protection of 57% (Figure 2a).

Next, we asked if the neuroprotective effect of enhanced RAS signaling could be mimicked by directly inhibiting the pl-VDAC-1 channel in wild-type cortical neurons via the extracellular application of mono-clonal anti-VDAC-1 antibody targeting the N-terminus of VDAC-1 [30,31,32]. Therefore, wild-type and synRas primary cortical cultures were preincubated with anti-VDAC-1 one hour before the excitotoxic stimulation with 1.0 mM glutamate for 60 min. After an additional 24 h of incubation, degenerated neurons were quantified by trypan blue exclusion assay. The number of degenerated neurons in control cells without any treatment or just with antibody incubation ranged from 18.7 ± 6.0%–21.6 ± 5.2% for wild-type neurons as well for synRas-derived neurons, respectively (Figure 2b). Stimulation with 1.0 mM glutamate resulted in 47.8 ± 8.9% degenerated wild-type neurons and 20.2 ± 5.8% degenerated synRas-derived neurons. Preincubation with anti-VDAC-1 antibodies followed by 1.0 mM glutamate treatment led to 25.6 ± 9.3% degenerated neurons in wild-type cultures and 22.0 ± 5.7% degenerated neurons in the case of synRas derived cultures. This reduction in excitotoxic glutamate effect by 46.4% clearly confirmed the involvement of pl-VDAC-1 in neuronal degeneration, and blocking of pl-VDAC-1 led to neuroprotection in wild-type neurons.

## 3. Discussion

Neurotrophins are extracellular factors that interact with their cognate tyrosine kinase receptors, thereby regulating neuronal survival, morphological differentiation, and synaptic connectivity in the brain [1,4]. As the intracellular membrane-anchored Ha-RAS proto-oncogene was previously identified as a major switch of neurotrophin-induced signal transduction [5], we here performed a proteome analysis of the brains of synRas transgenic animals expressing constitutively activated V12-Ha-RAS in neurons [9].

At first, we challenged the proteome data of synRas mice toward their relevance to explain the previously described phenotype concerning the modulation of synaptic function and connectivity [40]: an enhanced neurotransmitter release was associated with the accumulation of docked vesicles at the presynaptic membrane [41]. Notably, an enhanced synaptic long-term potentiation correlating with memory processes was sensitive to MEK inhibition by U0126, supporting a MAPK-dependent change in synaptic function in two independent animal models [40,42]. Using the proteome approach, we confirmed an increase in phosphorylated synapsin I at the MAPK phosphorylation target sites (sites 4/5 and 6), which was shown to regulate neurotrophin-induced synaptic release [43,44].

With these initial results, we were encouraged to search for the mechanism of the well-documented neuronal protection found in synRas transgenic mice focusing on the regulation of the VDAC-1 membrane protein (Table 1). 

### 3.1. Downregulation of pl-VDAC-1

Proteome or Western blot analysis did not allow for the discrimination between mt-VDAC-1 and pl-VDAC-1 because the amino acid sequence does not differ between both splice variants [45]. Using conventional subcellular fractionation methods, a downregulation of VDAC-1 was found in the mitochondrial fraction [39] (Table 1). However, due to the functional network between mitochondrial membranes, its associated endoplasmic reticulum, and the plasmalemmal membrane, the attempt to identify a differential localization of VDAC-1 depended on advanced preparation methods leading to the loss of functional properties [46]. Thus, we decided to analyze the endogenous mRNA splicing variants enabling specific targeting of the VDAC-1 proteins to either mitochondria or plasmalemmal membrane via a transmembrane signal peptide [25].

We here observed for the first time a selective decrease in mRNA level for pl-VDAC-1 in the hippocampus and cortex of adult synRas mice (Figure 1). Notably, in synRas-derived cortical cultures, the pl-VDAC-1 was also selectively downregulated on the mRNA level (Figure 1), correlating with decreased levels of NADH–ferricyanide reductase activity (Figure 1) at the cell surface as previously shown to be associated with pl-VDAC-1. Although the properties of the pl-VDAC-1 are not completely revealed, under healthy conditions, it contains an NADH–ferricyanide reductase activity at the cell surface. Moreover, under apoptotic conditions, pl-VDAC-1 becomes activated by channel opening and oligomerization [28,29,31]. The mt-VDAC-1 mRNA levels remained unchanged between wild-type and synRas-derived cortical cells (Figure 1). These data suggest that the transgenic activation of RAS in neurons selectively decreases pl-VDAC-1 mRNA, which is associated with a reduced level of pl-VDAC-1 protein. 

### 3.2. Regulation of Splicing by Constitutive Activation of V12-Ha-RAS?

Our finding that the neutralization of V12Ha-RAS transgene-induced MEK activity by U0126 completely reconstituted pl-VDAC-1 mRNA levels reaching those of normal wild-type cortical cultures (Figure 1) supports the hypothesis that RAS reduces pl-VDAC-1 expression by downregulating the alternative splicing of the VDAC-1 gene.

Examples of the RAS signaling-mediated regulation of alternative splicing events have been described, such as for protein tyrosine phosphatase CD45 after the activation of T-cells [47]. Similarly, nerve growth factor (NGF)-induced RAS activation was able to change the alternative splicing of the extracellular matrix protein Agrin in PC12 cells [48]. Furthermore, a well-studied system is the glycoprotein CD44, in which up to 10 different exons can be integrated by alternative splicing. T-cells express CD44 on the cell surface. The integration of exon v5 into CD44 is mediated by a complex of the splicing factor Sam68 (Src-associated in mitosis) and a co-splicing factor SRm160 (serine/arginine repeat-related nuclear matrix protein of 160 kDa), but Sam68 is dependent on activating phosphorylation by RAS/ERK activity [49,50,51]. In carcinoma cells of prostate epithelial cells, a reduced pl-VDAC-1 expression was reported [52], while an increased pl-VDAC-1 expression was found in human pancreatic carcinoma cells [53]. Here, we show a downregulation of an alternatively spliced mRNA by oncogenic V12-Ha-RAS for the first time (Figure 1). 

### 3.3. Cortical synRas-Derived Cultures Show Protection against Excitotoxic Glutamate 

Neuroprotection by constitutive activation of RAS in synRas mice was demonstrated not only for dopaminergic neurons in the substantia nigra but also for a variety of mechanical or chemical brain injuries in previous publications [9,13,14,15,38,40]. As in Alzheimer’s and Parkinson’s disease and many unrelated disorders in neuronal injury, the final common pathway leads to neuronal cell death, which we challenged by glutamate excitotoxicity [54,55], and we clearly demonstrated here glutamate-dependent protective effects by the V12-Ha-RAS transgene in cortical cultures (Figure 2). Glutamate binds to α-amino-3-hydroxy-5-methyl-4-isoxazolepropionic acid (AMPA-) and N-methyl-D-aspartate (NMDA) receptors, leading to a massive influx of Na^+^ and Cl^−^ -ions, resulting in a depolarization of the plasma membrane which causes the opening of Ca^2+^ channels. A massive influx of extra-cellular Ca^2+^-ions and Ca^2+^ release out of the mitochondria leads to the activation of nitric oxide synthase (NOS), proteases such as calpain, and the release of apoptosis-inducing factor (AIF) starting apoptosis [56,57]. In primary cortical cultures, glutamate-induced apoptosis activates calpain as well as caspase-3 [58]. Recently, a quantitative proteomic analysis of primary cortical cultures showed that 100 proteins were changed regarding their phosphorylation level and abundance upon excitotoxic glutamate stimulation [59]. Accordingly, an important role of RAS, along with its downstream MAPK/ERK and PI3K/Akt signaling pathways for neuroprotection in glutamate excitotoxicity, was observed in this study [59]. Consistently, the pre-incubation of hippocampal neurons with BDNF protected against glutamate-induced apoptosis by activating the PI3K and RAS/MAPK signaling pathways [60]. The possible contributions between MAPK/ERK and PI3K/Akt signaling for protection were not further investigated here. 

### 3.4. Monoclonal Antibody-Mediated pl-VDAC-1 Channel Inactivation Protects from Excitotoxic Glutamate in Cortical Neurons

In order to test if the RAS protective effect could be mimicked by blocking the pl-VDAC-1, we applied an anti-VDAC-1 antibody with an epitope directed against the N-terminal 100 amino acids of VDAC-1 located within the channel pore but accessible from the extracellular side of the pl-VDAC-1 (Figure 1a). Here, we report that wild-type primary cortical neurons are protected by anti-VDAC-1 antibody to a similar extent as the V12-Ha-RAS transgene-derived neurons (Figure 2). Notably, the effects of pl-VDAC-1 channel blocking by monoclonal antibody could not further enhance the protection resulting from transgenic RAS-induced downregulation. These observations are compatible with the hypothesis that cortical neuron protection achieved either via transgenic RAS activity or by channel blockade of pl-VDAC-1 with extracellular antibody is based on a common or similar mechanism. 

The above results are in line with previous studies showing that the extra-cellular application of anti-VDAC-1 antibody prior to a pro-apoptotic stimulus prevented cells from undergoing apoptosis in cells of the mouse hippocampal line HT22, in the human neuroblastoma cell line SK-N-MC and in primary differentiated hippocampal neurons [30,31]. 

Furthermore, a single-channel pore opening of pl-VDAC-1 by staurosporin or channel closure by application of this monoclonal antibody, respectively, was previously demonstrated in isolated patch clamp experiments [30,31].

In addition, anti-VDAC-1 antibody promoted the cellular protection of amyloid β peptide-mediated cell death in the mouse cholinergic septal neuronal cell line SN56 and HT22 cells [31,61], and against 15-deoxy-Δ^12,14^-prostaglandin J2 (15d-PGJ_2_)-mediated cell death in rat primary cortical neurons [32].

### 3.5. Pl-VDAC-1 in Neurodegenerative Disease

Pl-VDAC-1 levels are elevated in the cerebrospinal fluid of Alzheimer’s patients as a component of the ER signalosome together with caveolin-1, flotilin-1, and estrogen receptor alpha (Erα), insulin growth factor-1 receptor β (IGF1Rβ), and prion protein (PrP) [36]. The pl-VDAC-1 interacts with estrogen receptor α (mERα), and this complex modulates the amyloid beta (Aβ)-mediated neurotoxicity [61]. In a special form of lipid rafts called caveolae, pl-VDAC-1 was accumulated, and its interaction with mERα was altered in the human cortex and hippocampus derived from patients suffering from Alzheimer’s disease (AD) compared to non-pathological samples [33]. Furthermore, Aβ leads to the dephosphorylation of pl-VDAC-1 in lipid rafts, resulting in cell death, which was confirmed in lipid rafts in brains obtained from AD patients [62]. Both pl-VDAC-1 and mt-VDAC-1 are involved in Aβ-mediated neurotoxicity according to the following model: Outside the cell, an electrostatic interaction between Aβ with the pl-VDAC-1 results in a heterologous oligomer between pl-VDAC-1 and Aβ enabling Aβ to pass into the neuron. After translocation into the cytosol, an interaction between Aβ and mt-VDAC-1 occurs, leading to the detachment of hexokinase-1 from mt-VDAC-1. This allows the formation of a hetero-oligomer between mt-VDAC-1 and Aβ, thereby releasing cytochrome c into the cytoplasm, which promotes apoptotic cell death [34,63]. Recently, VBIT-4 was developed as an inhibitor for VDAC-1, preventing the oligomerization of VDAC-1 and the formation of the heterologous oligomer between VDAC-1 and Aβ, making this inhibitor a potential drug candidate for the treatment of AD [64].

In a cellular model of Parkinson’s disease, the dopaminergic cell line SH-SY5Y was exposed to the mitochondrial complex-1 inhibitor rotenone leading to increased VDAC-1 levels [65], while in post-mortal brain tissue, VDAC-1 levels are clearly reduced [35]. A potential involvement of VDAC-1 is also proposed for patients with autism where antibodies against the hexokinase-1 (HK-1) have been found [66]. The complex between HK-2 and VDAC-1 has been shown on a structural level to achieve a partial closure of the channel [67], thereby explaining the protective function of hexokinase against apoptosis. Thus, competing antibodies in the patient’s serum could release the neuroprotective function by hexokinase. Taken together, the possible role of pl-VDAC-1 in the etiology of PD still needs further investigation. 

## 4. Materials and Methods

### 4.1. Animals

All analyses were carried out using the transgenic mouse line NMRI-synRas-50 established in the Department of Molecular Neurobiochemistry, Ruhr-Universität Bochum, Germany. These animals have a bicistronic construct of the rat synapsin I promoter controlling the expression of the human G12V-Ha-RAS oncogene and lacZ gene connected via an internal ribosomal entry site (IRES) [9]. The results of comparison derive from using heterozygous synRas mice (constitutively activated G12V-Ha-RAS expressing selectively in neurons) with wild-type littermates (synRas negative) as controls. Animals had access to food and water ad libitum and were housed under a 12 h light and 12 h dark cycle. Mice were genotyped by PCR using the primers as indicated in the Supplementary Materials (Table S2). All procedures were conducted in agreement with the Animal Protection Law of Germany.

### 4.2. Preparation of Synaptosomal and Mitochondrial Fractions

Male mice (eight weeks after birth) were killed according to the guidelines of the Animal Protection Law of Germany and the European Communities Council Directive (1986/86/609/EEC), and the brains were removed after decapitation. The cortices and the hippocampi were immediately frozen in liquid nitrogen. The synaptosomal and mitochondrial fraction of the murine cortex and hippocampus were prepared as previously described [39]. All steps were performed at 4 °C. Homogenates were prepared from frozen cortices or hippocampi by lysis in ice-cold Buffer A (5 mM HEPES, pH 7.4, 1 mM MgCl_2_, 0.5 mM CaCl_2_, 40 mM NaF, 1 mM Na_3_VO_4_, 0.1 mM phenylmethylsulfonylfluoride, 1 µg/mL of aprotonin, 1 µg/mL of leupeptin, 1 mM benzamidine, 0.1 mM pepstatin and phosphatase inhibitor mixture I (Sigma-Aldrich, Steinheim, Germany) with glass homogenizer (12 strokes). Brain extract homogenates were centrifuged at 1400× *g* for 10 min. While the supernatant (S1) was saved, the pellet (P1) underwent further homogenization via a glass homogenizer (5 strokes) and centrifugation at 700× *g*. The obtained supernatant (S1′) was pooled with S1. The combined supernatants S1 and S1′ were centrifuged at 13,800× *g* for 10 min to collect the pellet (P2). The supernatant (S2) was used as a cytosolic fraction. Buffer B (0.32 M sucrose, 6 mM Tris, pH 8.0, 40 mM NaF, 1 mM Na_3_VO_4_, 0.1 mM phenylmethylsulfonylfluoride, 1 µg/mL of aprotonin, 1 µg/mL of leupeptin, 1 mM benzamidine, and 0.1 mM pepstatin) was used for resuspending pellet P2. A discontinuous sucrose gradient (0.85 M/1 M/1.15 M sucrose solution in 6 mM Tris, pH 8.0) was loaded by the P2 suspension and centrifuged for 2 h at 82,500× *g* in a SW-41 rotor (Beckman Coulter, Brea, CA, USA). A syringe needle was used for collecting the synaptosomal fraction, which was located between 1 M and 1.15 M sucrose. The mitochondrial fraction, which contains the attached endoplasmic reticulum and plasmalemma membrane, was collected as a pellet (P3) after the last centrifugation [46]. 

### 4.3. 2D-Difference Gel Electrophoresis (DIGE)

The mitochondria and the synaptosomes obtained from four wild-type and four synRas brain cortices were resuspended in solubilization buffer (7 M urea, 2 M thiourea, 20 mM DTT, 1% ampholine 3.5–10, 4% CHAPS, 1% Nonidet P-40 (NP-40)) and sonicated in an ice-cooled ultrasonic bath for 10 min. The proteins were purified from salts and lipids using precipitation by methanol in the presence of chloroform. Overall, 150 µg of every purified protein mixture was dissolved in labeling buffer (7 M Urea; 2 M Thiourea; 20 mM Tris-HCl pH 8.5; 4% CHAPS; 1% NP-40), and each sample was labeled separately using a CyDye DIGE Fluor (Cy3 for WT and Cy5 for synRas, respectively). The wild-type samples were then pooled with corresponding synRas samples (ampholine up to 1% and DTT up to 20 mM were added) and loaded onto an isoelectric focusing (IEF) gel. Gel composition for IEF contained 8 M urea, 4% acrylamide/bis-acrylamide (32:1), 1.5% CHAPS, 0.5% NP-40, 2% ampholine 3.5–10, 3% ampholine 5–8, 0.02% APS and 0.05% TEMED, in tubes with a 1.5 mm diameter and a 170 mm length. Cathode and anode buffers were 50 mM NaOH in H_2_O and 6 mM H_3_PO_4_, respectively. Samples were applied to the basic end of the tube gel and run on a PROTEAN II xi 2D Cell (Bio-Rad Laboratories, München, Germany); voltage profile: 100 V for 1 h, 200 V for 1 h, 300 V for 1 h, 400 V for 1 h, 500 V for 1 h, 600 V for 1 h, 700 V for 10 h, 900 V for 0.5 h (room temperature). Prior to SDS-PAGE, gel tubes were equilibrated for 15 min in 15 mL equilibration buffer (50 mM Tris-HCl pH 6.8, 6 M urea, 30% glycerine, 2% SDS) containing 30 mM DTT, followed by a second 15 min equilibration step in 15 mL equilibration buffer (see above) with 2% iodoacetamide and 200 mL saturated bromophenol blue solution. SDS-PAGE as a 2-D separation was performed at room temperature on gradient polyacrylamide gels (9–16% acrylamide, 160 × 180 × 1.5 mm, PROTEAN II xi Multi-Cell, 40 mA per gel for 4.5 h). Prepared 2D-gels have been imaged using the Typhoon 9400 Variable Mode Imager (Amersham—GE Healthcare, Freiburg, Germany) followed by analysis with ImageQuant 5.2/DeCyder 6.5 software (Molecular Dynamics—GE Healthcare, Freiburg, Germany). A match set consisting of eight images, four for synRas, and four for WT samples was created, and one image from the WT sample was defined as the match set standard used for spot matching. For the quantification and normalization of the protein abundance of detected spots, OD values of individual spots were divided by the total OD values of all spots present in the images and expressed in %vol. The differential in-gel analysis was used for intra-gel spot detection and quantification. Protein spots with an expression level greater than 1.5-fold change between wild-type and synRas samples (significance at least *p* < 0.05 evaluated by Student’s *t*-test) were defined as being differentially expressed. These statistical calculations were carried out using Microsoft Excel (Redmond, WA, USA). 

### 4.4. Matrix-Assisted Laser Desorption/Ionization—Time of Flight (MALDI-TOF)

For visualization, the polyacrylamide gels were washed, stained by the silver/thiosulfate technique, and scanned. Spots of interest were manually excised and further processed by modified porcine trypsin (Promega, Mannheim, Germany) digestions as previously published [68]. An amount of 1 µL of protein proteolytic fragment solution in 0.1% trifluoroacetic acid was mixed on the MALDI target with 1 µL 2,5-dihydroxybenzoic acid matrix solution (50 mM in 20% acetonitrile and 0.1% trifluoroacetic acid) and air dried. A MALDI-TOF-mass spectrometer Reflex III (Bruker Daltonics, Bremen, Germany), which was equipped with a 337 nm UV laser, was used for obtaining mass spectra by registering positive ions. Reflection mode was used for obtaining mass spectra of digests at a mass accuracy of less than 0.01%, and additional calibration was performed for the peaks of trypsin autolysis. Proteins were identified by PMF with MASCOT software (Matrix Science, Boston, MA, USA) utilizing the NCBI database according to the following parameters: 1–2 missed cleavages, trypsin enzyme, variable modifications by oxidation (M) and propionamide (C) and mass tolerance of 150 ppm.

### 4.5. Western Blot

In the case of cortical and hippocampal lysates, the tissue was homogenized in buffer A (see Section 4.2) using a glass homogenizer (12 strokes). In the case of mitochondrial fraction, the pellet of mitochondria (P3) was resuspended in buffer A. Then, the tissue homogenate or suspension of synaptosomes or mitochondria were mixed with an equal volume of 2× Lysis buffer (200 mM NaCl, 100 mM Tris-HCl pH 7.4, 2% Triton X-100, 1% NP-40, 30 mM MgCl_2_, 0.2% SDS, 0.2% Sodium Deoxycholate, 80 mM NaF, 2 mM Na_3_VO_4_, 2× Complete Protease Inhibitor Cocktail (Roche, Mannheim, Germany)) and incubated at +4 °C for 10 min. Protein concentration was measured using DC protein Assay (Bio-Rad), with bovine serum albumin as the standard. Overall, 10–40 µg of protein sample from 4 to 7 biological replicates (either individual samples or pooled from each animal group) were subjected to SDS-PAGE (12% acrylamide, Protean II system, Bio-Rad) and transferred onto 0.45 µm nitrocellulose membranes (Protran BA85, Whatman, Germany) using the tank transfer system (Bio-Rad). Immunoblots were performed by incubation 1–2 h at room temperature or overnight at +4 °C with the primary antibody (Table S3). In preliminary experiments, all antibodies were proven to provide a specific band corresponding to the target protein with the correct molecular weight. The secondary antibody (Table S4) was incubated at room temperature for 2 h. Immunoreactivity of proteins was detected using the SuperSignal West Pico Chemiluminescent substrate (Pierce—Thermo Fisher Scientific, Waltham, MA, USA) followed by exposure to Hyperfilm-ECL film (GE Healthcare, Freiburg, Germany). The films were developed and scanned. The images were subjected to densitometry using TINA 2.0 Software (Raytest Isotopenmessgeräte GmbH, Sprockhövel, Germany, 1993). The intensity of all detected protein bands was normalized on the intensity of the loading control bands (tubulin). For each antibody/protein sample, we used two to four different gels.

### 4.6. Quantitative Real-Time PCR Analysis

Total RNA was isolated using the kit “Total RNA Isolation NucleoSpin RNA II“ (Macherey-Nagel, Düren, Germany) according to the manufacturer’s manual. Quantitative real-time PCR was performed using 50–100 ng RNA along with the one-step kit “QuantiTect^®^ SYBR^®^ Green RT-PCR Kit” (Qiagen, Hilden, Germany) following the manufacturer’s protocol on a LightCycler system (Roche). Each sample was run in duplicates. In all cases, lamin mRNA was used as a housekeeping gene quantified by specific primers (Table S5). The mt-VDAC-1 mRNA, as well as the pl-VDAC-1 mRNA, were analyzed using splice-variant specific primers (Table S5). The primer pair “pl-VDAC-1 q for” and “pl-VDAC-1 q rev” was designed to amplify a cDNA fragment of the pl-VDAC-1 as the primer “pl-VDAC-1 q for” hybridize to the sequence of the hydrophobic signal peptide of 13 amino acids which was shown by Buettner et al. [27]. The obtained crossing points (CP) were used in the calculations to compare the gene of interest normalized to the housekeeping gene according to the ΔΔCP methods [69]. The identity of each amplified DNA fragment was confirmed by subcloning into the vector pGEM T easy (Promega) and subsequent sequencing.

### 4.7. Primary Cortical Cultures

Cortical cultures using cortices of P0–P2 newborn mice of the mouse line NMRI-synRas-50 were prepared as previously described [70]. The cortices from each animal were processed individually in all steps because the genotype was unknown at the moment of preparation. Briefly, the cortices of each P0–P2 newborn NMRI mice were minced in ice-cold MPBS+/+ (modified phosphate-buffered saline supplemented with 0.25 mM CaCl_2_, 5.8 mM MgCl_2_, 10 mM HEPES, 1 mM sodium pyruvate (Sigma-Aldrich), 6 µg/mL DNase1 (Sigma-Aldrich), 1 mg/mL bovine serum albumin (BSA) (Sigma-Aldrich), 10 mM glucose, 25 U/mL penicillin, 25 µg/mL, streptomycin, 2 mM glutamine, 5 mg/mL phenol red (Sigma-Aldrich), and 4 mM NaOH) and digested with 10% trypsin (GE Healthcare) in MPBS−/− (MPBS+/+ without CaCl_2_ and MgCl_2_) for 15 min under shaking at 37 °C. After settling of the tissue, the supernatant (containing dissociated cells) was diluted two-fold with RPMI/10% FCS (2 mM glutamine, 10% FCS, 25 U/mL penicillin, 25 µg/mL, streptomycin, 0.00375% insulin (Sigma-Aldrich), 5 mM glucose, and 10 mM HEPES, in RPMI 1640 (Sigma-Aldrich)) to terminate digestion. Residual tissue pieces were dissociated by trituration in MPBS−/− with a cut plastic pipette tip three times, and both cell suspensions were pooled. The cells were collected by centrifuging at 200× *g* for 10 min while the centrifuge was cooling down from room temperature to 4 °C. The cell pellet was dissociated in RPMI/10% FCS and preplated on uncoated 60 mm plastic dishes (Sarstedt, Nümbrecht, Germany) for 1–3 h at 37 °C and 5% CO_2_. The neuron-enriched supernatant was collected and seeded at a density of 400,000 cells per 35 mm poly-L-ornithine-coated culture dish (Nunc—Thermo Fisher Scientific). Per newborn animal, one up to four culture-dishes were obtained. The following day, the media was exchanged against neurobasal medium (Invitrogen—Thermo Fisher Scientific, Waltham, MA, USA) containing B-27 serum-free supplement (Invitrogen—Thermo Fisher Scientific) (1× B27, 2 mM glutamine, 100 U/mL penicillin, 100 µg/mL streptomycin in neurobasal medium). Every second to the third day, the media was exchanged against fresh pre-incubated medium. The cells were used for experiments after 7 days in vitro. For quantitative real-time PCR analysis, two 35 mm culture dishes with 400,000 cells each were pooled for RNA isolation per condition.

### 4.8. Excitotoxic Glutamate Stimulation of Primary Cortical Cultures

The excitotoxic glutamate stimulation was performed as described elsewhere [70]. In short, the day before the glutamate stimulus, the media were replaced by NB/B27-, which had the same composition as NB/B27 but the B27 supplement in its antioxidant-free version (Invitrogen—Thermo Fisher Scientific). In some experiments, the cells were pre-incubated with anti-VDAC-1 (Sigma-Aldrich, order number: WH0007416M5-100UG) antibodies 1:1000 diluted in NB/B27- for 1 h before the glutamate stimulation. Prior to the glutamate stimulation, the cells were washed with PBS three times to remove traces of magnesium. The cells were treated with a balanced salt solution (BSS: 130.0 mM NaCl, 5.4 mM KCl, 1.8 mM CaCl_2_, 5.5 mM glucose, 20.0 mM HEPES, pH 7.4) which was supplemented with 10 µM glycine as well as 0.5 mM or 1.0 mM glutamate (Sigma-Aldrich) and incubated for 90 min or 1 h, respectively, at 37 °C and 5% CO_2_. After this stimulation, the solution was exchanged by NB/B27-, and the cells were cultivated for further 24 h at 37 °C and 5% CO_2_. PBS supplemented with 4% paraformaldehyde was used for fixing the cells, and nuclei were stained by 1 µg/mL Bisbenzimide H 3342 (Fluka, Buchs, Switzerland) in PBS. Degenerated neurons were quantified by scoring the percentage of neurons showing condensed or strongly fragmented nuclei as analyzed on an Olympus IX51 fluorescence microscope (Olympus, Hamburg, Germany). Alternatively, the number of degenerated neurons was determined using the trypan blue exclusion assay by exchanging the medium with a 1:6 mixture of 0.04% trypan blue (Sigma-Aldrich) in PBS. Degenerated neurons were stained in dark blue and counted correspondingly in ratio to the total number of neurons per field of view on a microscope (Olympus IX51). Per condition and experiment, 4–7 fields of view were analyzed. 

### 4.9. NADH–Ferricyanide Reductase Activity Derived from Primary Cortical Neuronal Cultures

The NADH–ferricyanide reductase activity was measured with minor modification, as recently published [31]. The medium of the cells was exchanged against 1 mL buffer (50 mM Tris-HCl pH 8.0, 500 μM β-NADH) providing 500 μM potassium ferricyanide (III) (K_3_Fe(CN)_6_) (Riedel-de-Haen—Honeywell Specialty Chemicals Seelze GmbH, Seelze, Germany) per 35 mm dish and the cells were incubated for 60 min at 37 °C and 5% CO_2_. Ferricyanide was reduced to ferrocyanide. Subsequently, an aliquot of the buffer was drawn to measure the absorbance at 420 nm with a photometer (UV1202, Shimadzu, Kyoto, Japan). The NADH–ferricyanide reductase activity was expressed as reduced ferricyanide in nmol/min/4 × 10^5^ cells. Per the experiment, each condition was measured in duplicates.

### 4.10. Partial Inhibition of MAPK Pathway

For the partial inhibition of MAPK signaling in primary cortex cultures, a medium change was performed using NB/B27 supplemented with 10 μM of the MEK1/2 inhibitor U0126 (Sigma-Aldrich) [40,71]. As a control, cells were cultured in NB/B27 supplemented with DMSO. This type of medium change was repeated daily for the following two days. 

### 4.11. Statistical Analysis

If not stated otherwise, the statistical significance was calculated using either unpaired two-tailed *t*-test or one-way analysis of variance (ANOVA) followed by Tukey post hoc test with the software GraphPad Prism version 10.1.0 for Windows (GraphPad Software, Boston, MA, USA, www.graphpad.com (accessed on 24 October 2023)). Statistical significance was given when the *p*-value was below 0.05 (*: *p* < 0.05, **: *p* < 0.01, ***: *p* < 0.001, ****: *p* < 0.0001).

## 5. Conclusions

**Figure 3:**
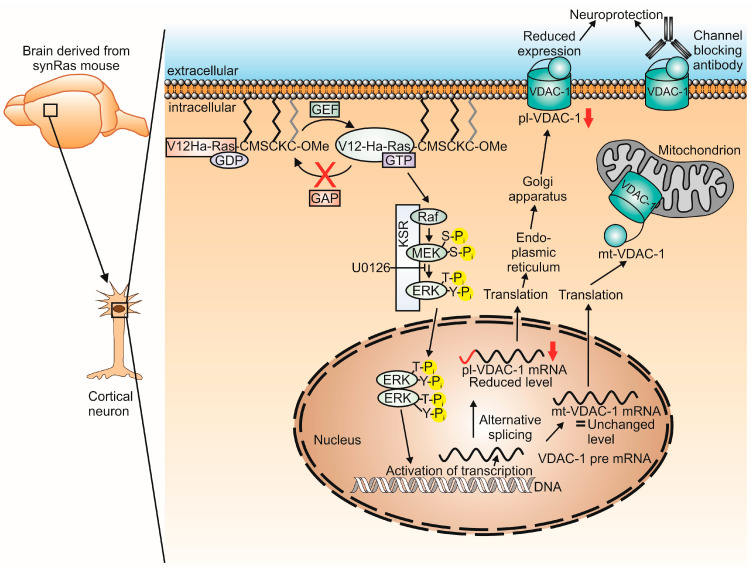
Activated RAS via the MAPK pathway influences the alternative splicing of VDAC-1 mRNA, leading to a reduced pl-VDAC-1 expression, which might be involved in synRas-mediated neuroprotection. V12Ha-RAS is shown schematically with some amino acids of the C-terminus in the one-letter code, demonstrating how RAS is anchored to the membrane by a Farnesyl (gray) and two Palmitoyl anchors (black). Once V12-Ha-RAS is activated in the GTP-bounded state, the V12 mutation inhibits its inactivation via GAP. A kinase suppressor of RAS (KSR) functions as a molecular scaffold for the kinases Raf, MEK, and ERK for effective signaling. Yellow circles with P_i_ indicate the phosphorylation of MEK at two serine residues and of ERK at one threonine and one tyrosine residue. U0126 inhibits MEK and thereby blocks any further downstream signaling. Activated ERK translocates into the nucleus and activates the transcription of certain genes. The VDAC-1 pre mRNA becomes alternatively spliced, leading to an unchanged level for mt-VDAC-1, while the pl-VDAC-1 mRNA is reduced in synRas mouse-derived cortical neurons. The sequence coding for the signal peptide is schematically highlighted in red in the pl-VDAC-1 mRNA. After translation, the mt-VDAC-1 is placed into the outer mitochondrial membrane, while the pl-VDAC-1 protein is transported via the endoplasmic reticulum and the Golgi apparatus and inserted into the plasmalemma. The VDAC-1 is schematically shown with a chain and ball motif representing the opening state: while the mt-VDAC-1 is open for metabolic exchange between the mitochondrion and the cytosol [72], the pl-VDAC-1 is closed under healthy conditions (as shown above) or open under apoptotic conditions (not shown above) [31]. In cortical neurons of the synRas mouse, the reduced expression of pl-VDAC-1 leads to neuroprotection similar to the extracellular application of an anti-VDAC-1 antibody blocking the pl-VDAC-1.

Starting out from work on peripheral neurons and transgenic mice showing that enhanced Ha-RAS-signaling in brain neurons may be considered as an intracellular replacement for neurotrophins [5,9], we here suggest that the downregulation of VDAC-1 by neuronal activation of RAS activity is a common denominator of neuronal protection for the following reasons: (i) VDAC-1 is downregulated in transgenic animals expressing constitutively activated (oncogenic) RAS in neurons, (ii) downregulation is restricted to plasmalemma (pl-)VDAC-1 while mitochondrial (mt-)VDAC-1 levels are not changed (Figure 3), (iii) the neuroprotective effect of RAS transgene signaling is fully prevented by the application of U0126 MEK inhibitor, and (iv) the extracellular application of channel-blocking monoclonal antibody against VDAC-1 localized at the cell surface mimic the protective effects achieved by enhanced intracellular RAS signaling in transgenic cortical neurons. Although we cannot exclude that other neuronal protection mechanisms exist, the results support the relevance of plasmalemma localized VDAC-1 as a target for optimizing neuroprotective approaches in clinical settings of transplantation and neurodegenerative diseases that are based on already available function-blocking monoclonal antibody.

## Figures and Tables

**Table 1:** Alterations in protein expression levels revealed by two-dimensional difference gel electrophoresis (2D-DIGE). Spots named M, S, and C correspond to mitochondrial, synaptosomal, and cytosolic fractions, respectively [39].

Name of Protein	Spot	Alteration synRas vs. WT, Fold	GenBank No.	MW, kDa	pI	Mascot Score	Function
**Upregulated proteins**							
ATP synthase, H^+^ transporting, mitochondrial F1 complex, *Subunit β*	M257	+3.3 ± 1.1	gi|28302366	56.3	5.19	293	ATP synthesis
29 kDa Fragment (central ATP-binding non-catalytic domain), ATP synthase, H^+^ transporting, mitochondrial F1 complex, *Subunits α*/*β*	M219, M220, M221	Appearance	Fragment of gi|28302366 and gi|15928789	On gel ~29.0	~6.0 - 8.0	79	Probably resulted in proteolysis of Full-length alpha or beta subunits (59/56 kDa)
ATP synthase, H^+^ transporting, mitochondrial F1 complex, *Subunit γ*	M246	+2.74 ± 0.74	gi|14715071	32.8	9.06	139	ATP synthesis
26 kDa fragment of ubiquinol-cytochrome c reductase core protein 1	M248	+3.4 ± 1.1	Fragment of gi|46593021	~26.0	5.81	229	aerobic respiration, oxidative phosphorylation
Cytochrome C-1	M247	Mitochondrial +2.2 ± 1.0	gi|13542841	35.3	7.12	85	mitochondrial respiratory chain
hypothetically predicted HAD hydrolase	M231	+1.30 ± 0.25	gi|13097531	28.0	6.31	78	oxidative phosphorylation
Amphiphysin	S1	+3.1 ± 0.5	gi|32451969	127.9	4.56	118	clathrin-mediated endocytosis, synaptic vesicle recycling
Dynamin	S4	+1.7 ± 0.3	gi|21961254	97.8	7.61	69	vesicle-mediated transport
Serpin b1a	C113	+2.5 ± 1.1	gi|12843390, gi|15029834	42.5	5.85	69	protein catabolism
**Downregulated proteins**							
Stathmin	C120	−9.1 ± 3.3	gi|14625464, gi|32449851	17.2	5.95	91	microtubule remodeling
Voltage-dependent anion channel 1 *[VDAC-1*, *porin-1]*	M216, M217	−2.9 ± 0.4 −2.2 ± 0.3	gi|1072040, gi|1098623, gi|6755963	32.3	8.55	116, 147	ATP release from mitochondrion to cytoplasm Participate in apoptosis
Oxoglutarate dehydrogenase *[OGDH, α-ketoglutarate dehydrogenase]*	S3	−1.30 ± 0.20	gi|15489120	116.4	6.36	96	TCA cycle
**Modified proteins**							
ATP synthase, H^+^ transporting, mitochondrial, delta subunit	S27	Shift upward	gi|16741459	18.7	5.03	77	ATP synthesis
Isocitrate dehydrogenase 3, β subunit	M244, M245	Redistribution of isoforms: +1.7 ± 0.4 −2.2 ± 0.5	gi|14290508	42.2	8.76	182	TCA cycle
Electron transferring flavoprotein, α polypeptide *[Alpha-ETF*, *MADD*, *GA2]*	M242, M243	Redistribution of isoforms: +1.4 ± 0.25 −2.8 ± 0.9	gi|31981826	34.9	8.62	252	oxidative phosphorylation
Vesicle-fusing ATPase [NSF; N-ethylmaleimide sensitive fusion protein]	C1–C4	Translocation from membranic fraction to the cytosol in Cytosol +2.60 ± 0.8	gi|31543349	82.6	6.55	208	vesicle-mediated transport
Vesicle-fusing ATPase [NSF; N-ethylmaleimide sensitive fusion protein]	S101–S104	In Synaptosomes −2.7 ± 0.6	gi|31543349	82.6	6.55	167	vesicle-mediated transport
Aconitase 2 (iron regulatory protein 1),	C26, C29	Redistribution of isoforms: +1.6 ± 0.3 −1.7 ± 0.4	gi|18079339	85.4	8.08	126, 105	TCA cycle

## Data Availability

The analyzed datasets are available upon request from the corresponding author.

## Supplementary Materials

The supporting information can be downloaded at https://www.mdpi.com/article/10.3390/ijms25053030/s1.
