# Supplementary material for: Neuronal Protection by Ha-RAS-GTPase Signaling through Selective Downregulation of Plasmalemmal Voltage-Dependent Anion Channel-1
Source: Int J Mol Sci. 2024 Mar 6;25(5):3030. doi: 10.3390/ijms25053030 (PMC10932472; doi:10.3390/ijms25053030)
Supplement: Supplementary file 1 [file ijms-25-03030-s001.zip › ijms-2842996-supplementary.pdf]

Supplementary Materials

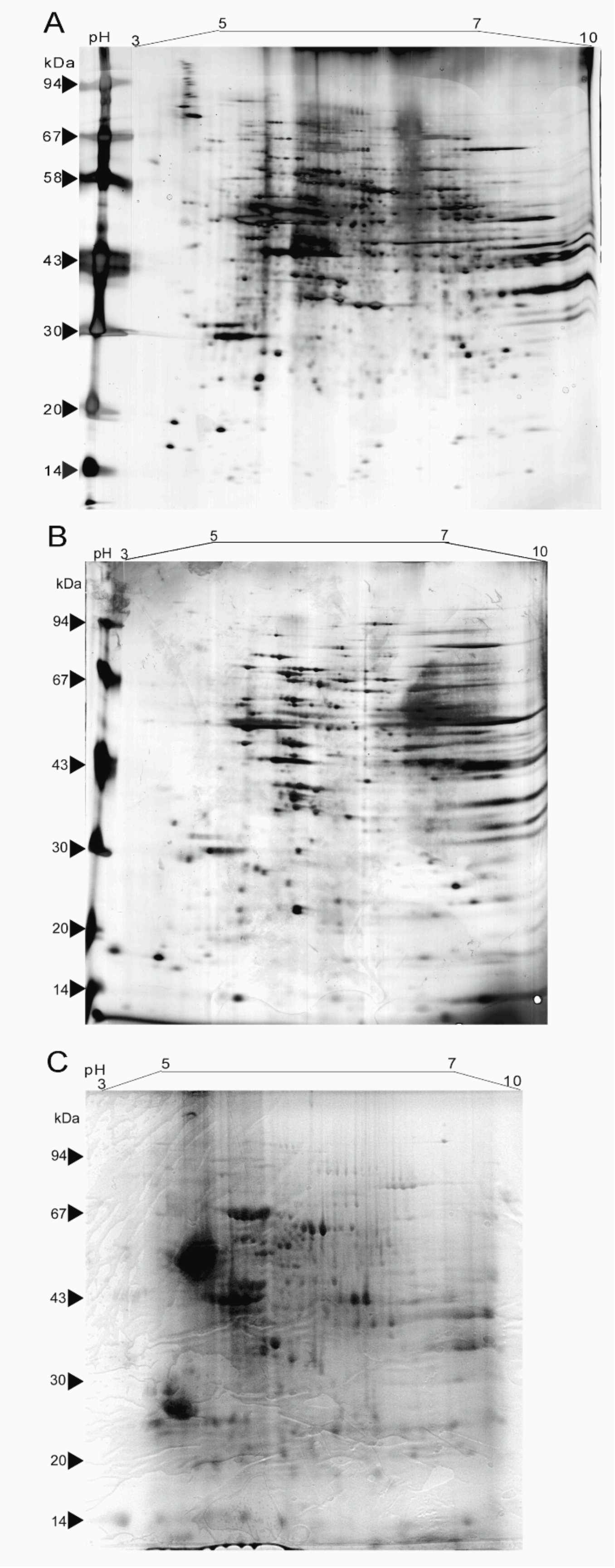

Figure S1. Representative silver/thiosulfate stained gel images of mouse brain proteomes fractions analysed by two-dimensional gel electrophoresis. Samples were pooled from WT and synRas homogenates. (A) Mitochondrial

fraction, (B) Synaptosomal fraction, (C) Cytosolic fraction. The first dimension was performed on pH 3–10 non-linear home-made isoelectric focusing (IEF) tube gels; the second dimension was run on 9-16 % acrylamide gel. These gels were further analysed for differential expression of the individual spots as described in the Materials and Methods. As one example, the same gel of the synaptosomal fraction (B) is shown in Figure S2 subjected to excitation of the fluorophores Cy3 / Cy5.

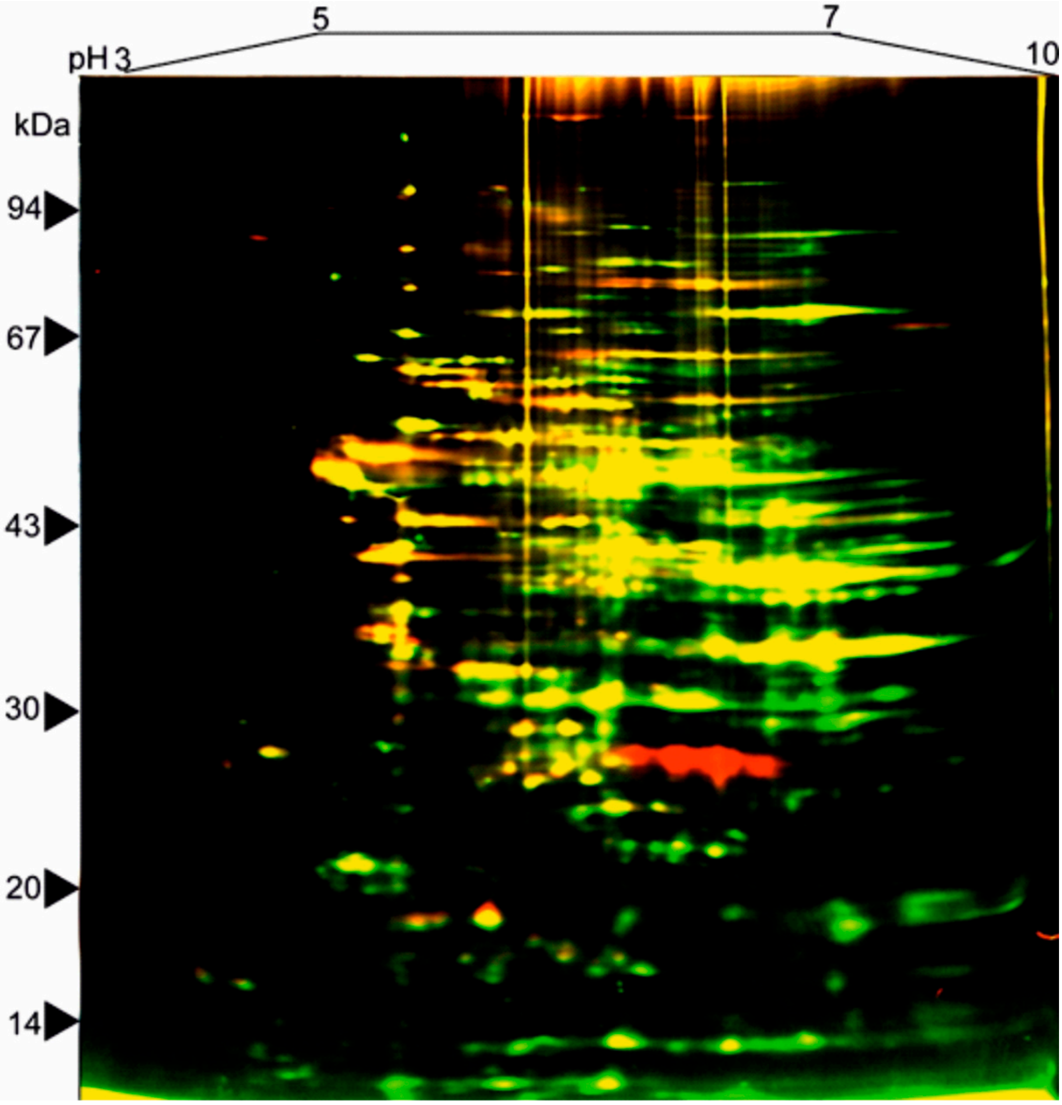

Figure S2. Overlap image obtained from the Two-Dimensional Difference Gel Electrophoresis (2D-DIGE) of pooled WT and synRas homogenates, as shown in Figure S1 B (synaptosomal fraction): first dimension: Ampholines 3-10, non-linear gradient. Second dimension: 9 - 16 % polyacrylamide gel. WT proteins are shown in green (Cy3), synRas proteins are shown in red (Cy5) upon excitation at 532 nm and at 633 nm, respectively. Yellow spots correspond to equal amount of proteins in both samples.

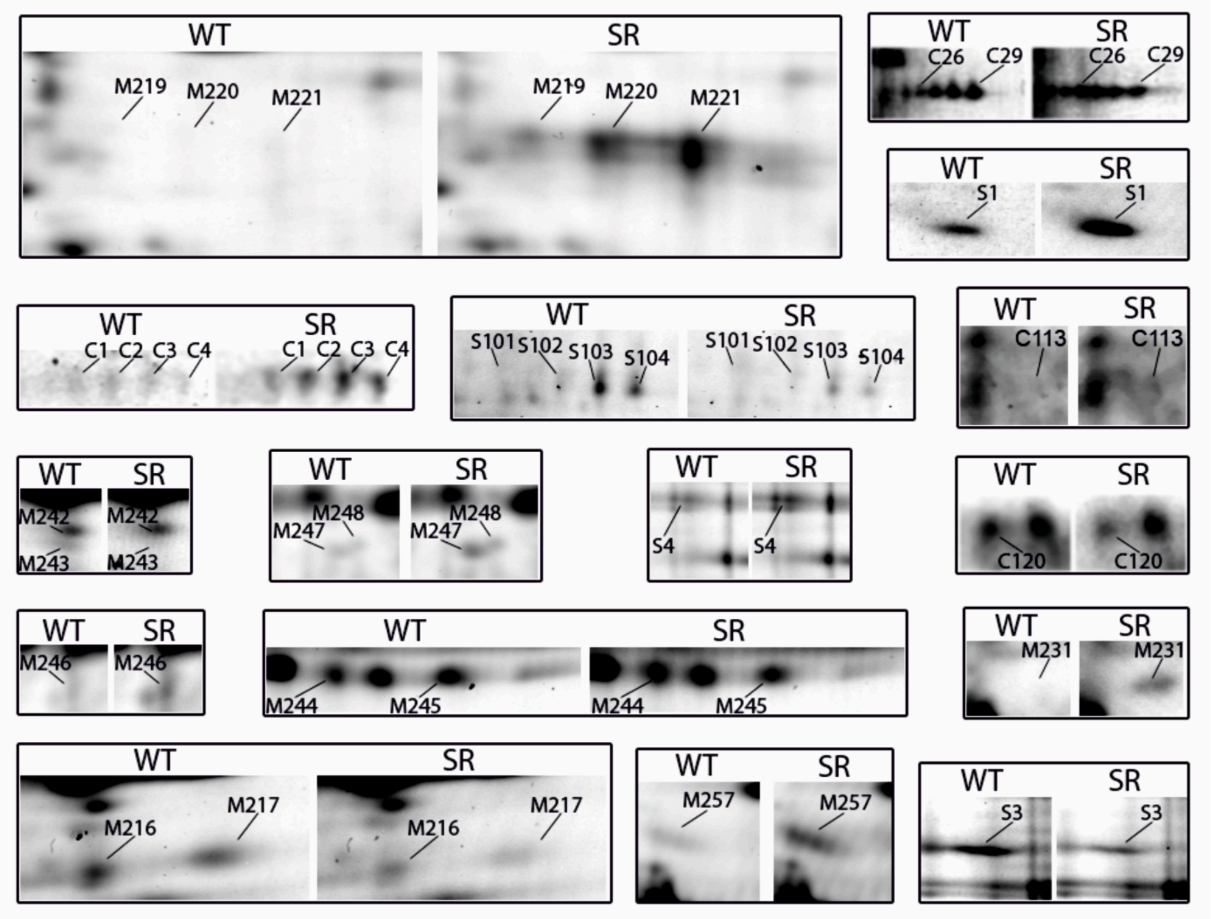

Figure S3. Details of 2D-DIGE gel images showing selected differentially regulated proteins. Representative partial 2-DE images of mouse brain cortices, showing differences in the protein expression at wild-type (WT) compared to synRas transgenic mice (SR). WT and SR images were resolved from the same gel using two-channel imaging on Typhoon 9400 imager (channel 1: Cy3, 532 nm excited fluorescence, and channel 2: Cy5, 633 nm excited fluorescence respectively). The proteins were separated as stated in Figure S1. Please note, the labelling of the spots indicates the fraction, as C represents the cytosolic fraction, M the mitochondrial one and S stands for the synaptosomal fraction. Furthermore, the same labelling is used in Table 1.

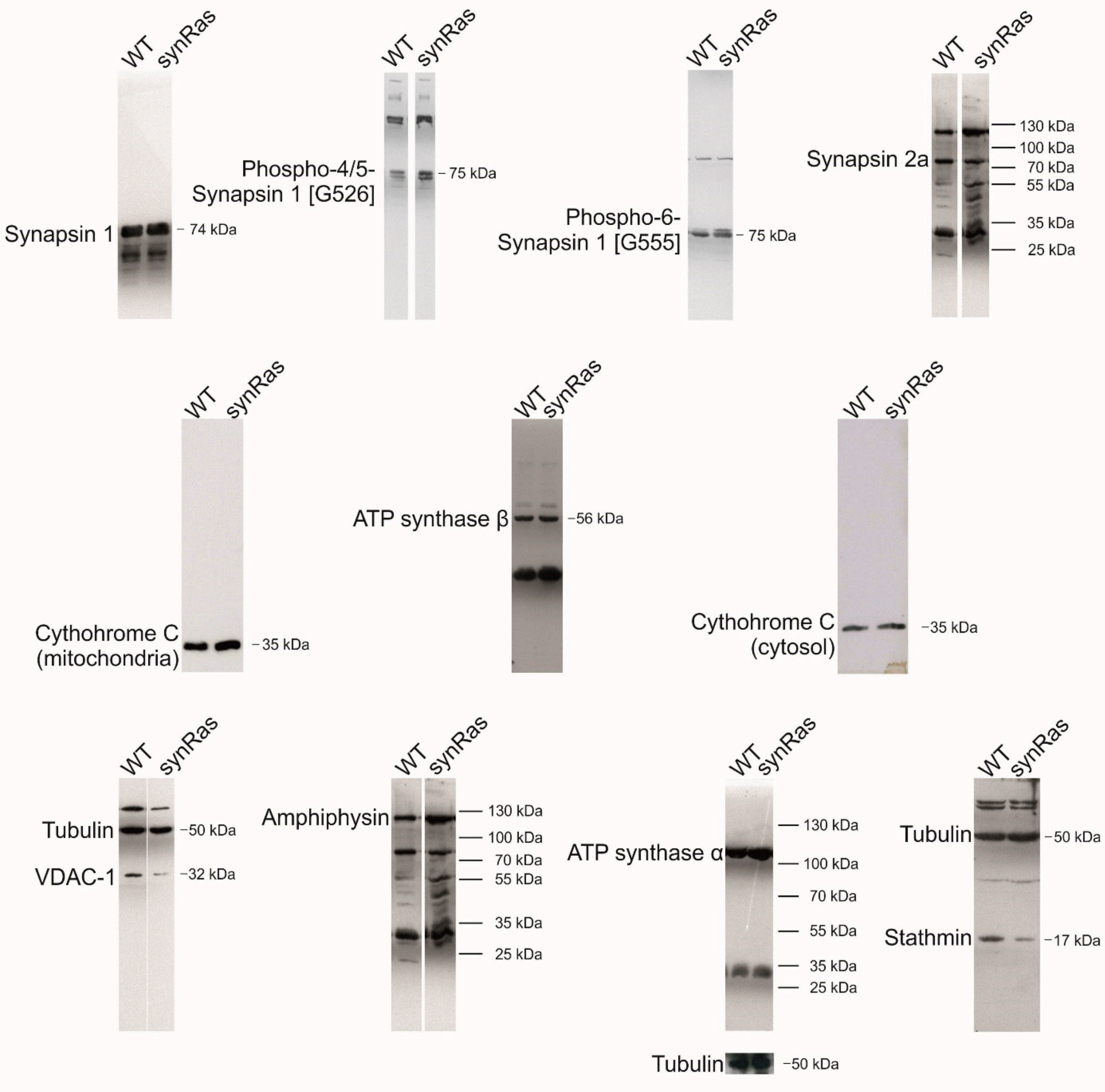

Figure S4. Western Blots for validation of differentially expressed proteins between wild type (WT) and synRas derived fractions. Representative images are presented. Blots were sequentially probed with several antibodies and we cannot completely exclude cross-reactivity. However in this case we would expect only minor reductions (if any) in the difference of the intensity of a given protein.

Table S1. Western blots of differentially expressed proteins between wild type and synRas derived fractions from the synaptosomal fraction were quantified using Tubulin for normalization and are expressed in fold changes ± SEM (*t*-test: NS: not significant, *:p<0.05, **:p<0.01).

Protein	Change in synRas, fold
Synapsin I	**-1.06 ± 0.16** (NS)
Phospho-4/5 synapsin I	**+1.66 ± 0.33** (*)
Phospho-6 synapsin I	**+1.63 ± 0.27** (**)
Synapsin IIa	**-1.43 ± 0.23** (NS)
ATP synthase (subunit β)	**+1.70 ± 0.14** (**)
VDAC	**-2.94 ± 0.39** (**)
Amphiphysin	**+1.32 ± 0.11** (*)
ATP synthase (subunits α)	**+1.30 ± 0.21** (NS)
Stathmin	**-2.44 ± 0.59** (*)

Table S2. Primers for Genotyping

Name	Sequence (5'→ 3')
Ras for	TGACCATCCAGCTGATCCAGAA
Ras rev	CTCCCCATCAATGACCACCTG

Table S3. Primary Antibodies

Antibody	Manufacturer	Order Number	Dilution
mouse anti-VDAC	Mitosciences, Eugene, OR, USA	MSA03	1:1000
mouse anti-VDAC-1	Sigma-Aldrich	WH0007416M5-100UG	1:1000
mouse anti-ATP synthase alpha subunit	Mitosciences	MS502	1:1000
mouse anti-ATP synthase beta subunit	Mitosciences	MS503	1:1000
mouse anti-cytochrome c	Mitosciences	MSA06	1:1000
rabbit anti-stathmin-1	Cell Signaling Technology, Danvers, MA, USA	#3352	1:800
mouse anti-synapsin-1	Synaptic Systems, Göttingen, Germany	#106001	1:1500
rabbit anti-amphiphysin	Calbiochem - Merck, Darmstadt, Germany	NB30T	1:1000
rabbit anti-phosphosynapsin 1 site 4/5	kindly provided by Prof. A. Nairn, Lab. Molecular and Cellular Neuroscience, Rockefeller University, New York, USA	G526	1:750
rabbit anti-phosphosynapsin 1 site 6	kindly provided by Prof. A. Nairn	G555	1:750
mouse Anti-β-Tubulin	Sigma-Aldrich	T8328	1:1000

Table S4. Secondary Antibodies

Antibody	Manufacturer	Dilution
horseradish peroxidase-linked anti-mouse IgG-POD	Dianova, Hamburg, Germany	1:3000
anti-rabbit IgG-POD	Dianova	1:8000

Table S5. Primers for Quantitative Real-Time PCR Analysis

**Name**	**Sequence (5'→ 3')**
Lamin B1 for	AGGATCCAGGAATTGGAGGAC
Lamin B1 rev	ATCACTCAGCTGCTGCTGCAT
mt-VDAC-1 q for	CCCACATACGCCGATCTTGG
mt-VDAC-1 q rev	GTGGTTTCCGTGTTGGCAGA
pl-VDAC-1 q for	TTCATTCTTTCTCGTGCTTTTG
pl-VDAC-1 q rev	CCGTTCACTTTGGTGGTTTC

All custom DNA oligonucleotides were synthesized by Sigma-Aldrich (Taufkirchen, Germany).
